# The Impact of the COVID-19 Pandemic on Educational and Academic Activities of Healthcare Professionals in Bahrain

**DOI:** 10.7759/cureus.50779

**Published:** 2023-12-19

**Authors:** Khadija Ali, Hasan M Isa, Maryam F Ali, Fatema A Ali, Zahra Alsahlawi, Hussain Alsaffar

**Affiliations:** 1 Department of Pediatrics, Salmaniya Medical Complex, Manama, BHR; 2 Department of Pediatrics, Arabian Gulf University, Manama, BHR; 3 Department of Child Health, Pediatric Endocrinology Unit, Sultan Qaboos University Hospital, Muscat, OMN

**Keywords:** bahrain, healthcare professionals, doctors, nurses, academic activities, educational activities, covid-19

## Abstract

Introduction: Since its emergence, the COVID-19 pandemic has had a radical effect on different aspects of health worldwide. The burden created by the COVID-19 pandemic on healthcare systems has also involved educational and academic activities among healthcare professionals.

Objectives: This study aimed to explore the effect of the COVID-19 pandemic on the participation of doctors and nurses in educational and academic activities in Bahrain.

Methods: A cross-sectional survey was conducted through an online questionnaire that was distributed among 204 healthcare providers (HCPs) in the Kingdom of Bahrain.

Results: Out of 204 HCPs, 110 (53.9%) were doctors and 94 (46.1%) were nurses. A total of 154 responses were received (100 (64%) doctors and 54 (35.1%) nurses) with an overall response rate of 75.5%. Seventy-four (74%) doctors and 32 (59.2%) nurses stated that their overall academic activities had decreased since the start of the pandemic, yet this was not statistically significant (P=0.059). Unlike nurses, doctors attended more webinars 54 (54%) and online courses 47 (47%), compared to the time before the pandemic (P=0.022, P=0.014, respectively).

Conclusion: The COVID-19 pandemic had a negative impact on educational and academic activities among healthcare workers. However, it created an opportunity to expand the use of electronic and online methods in those activities.

## Introduction

In March 2020, the World Health Organization announced the global pandemic of the novel COVID-19 as a rapidly spreading viral infection causing mainly respiratory distress syndrome [1]. In the Kingdom of Bahrain, the first reported case was detected in late February 2020, and the first case of COVID-19-related death was declared on March 16th, 2020 [2]. Ever since, the government of Bahrain has implemented several enhanced screening and quarantine measures to contain the spread of the virus [3]. The COVID-19 virus was widely recognized as a contagious and dangerous infectious disease. Studies have highlighted the contagious nature of COVID-19, with reports indicating that the virus is highly transmissible, leading to rapid spread. The transmission potential of COVID-19 has raised substantial concerns, especially within healthcare environments, where healthcare workers face an elevated risk of coming into contact with the virus. Moreover, the perilous characteristics of COVID-19 have been linked to its effects on susceptible demographic groups, including older adults and individuals with pre-existing health conditions, resulting in heightened anxiety and apprehension regarding the potential consequences of contracting the virus. The spread of the virus has brought significant implications for public health, healthcare practices, and individual behaviors.

Along with the worldwide actions to comply with the social-distancing measures, many educational institutes were closed, and a dramatic shift of educational activities to online digital platforms was observed [4]. The closure of educational institutions as a preventive strategy has been a topic of thorough investigation, with research endeavors delving into the correlation between closures and the impact of educational and research activities. It is noteworthy that the timing of these closures has been observed to align temporally with reductions in COVID-19 incidence and mortality, underscoring the potential influence of these measures on public health outcomes.

In Bahrain, the decision to close the educational institutes and universities was implemented on February 25th, and online learning was adopted [5,6]. Likewise, educational and academic activities for healthcare workers, including continuing medical education, which is of vital importance, were tremendously affected by the pandemic [7]. Accordingly, this study aimed to highlight the effect of the COVID-19 pandemic on the participation of doctors and nurses in educational activities in the Kingdom of Bahrain.

## Materials and methods

This is a prospective cross-sectional study where healthcare providers (HCPs) in the Kingdom of Bahrain, including doctors and nurses of different specialties, were asked to answer a questionnaire to assess their level of involvement in academic and educational activities during the COVID-19 pandemic in comparison to the period before the pandemic. An online questionnaire that consisted of eight closed-ended questions and was previously validated was used in this study. The details of the questionnaire are shown in Table 1.

**Table 1:** Questionnaire details

Please answer the following questions from the suggested answers							
What is your job description?	Doctor, nurse						
What is your job level?							
Consultant, specialist, fellow	Resident/trainee sister/staff nurse, other						
What is your specialty?							
Adult, pediatric	Both						
What is your subspecialty?							
Allergy and immunology, anesthesiology, cardiology, colon and rectal surgery, critical care, dermatology, developmental-behavioral pediatrics, diagnostic radiology, emergency medicine, endocrinology, diabetes, family medicine, gastroenterology, general medicine, general pediatrics, general surgery, geriatric medicine, hematology, hematology and oncology, infectious disease, internal medicine, interventional cardiology, medical genetics	Metabolism, neonatal-perinatal medicine, nephrology, neurology, nuclear medicine, obstetrics and gynecology, oncology, ophthalmology, orthopedic surgery, otolaryngology, pediatric surgery, pathology, plastic surgery, psychiatry, radiation oncology, respiratory, rheumatology, sports medicine, thoracic surgery, urology, vascular surgery, other (please specify)						
What is the number of the following activities during the COVID-19 pandemic?	0	1	2	3	4	5	>5
Completed original articles that you have submitted							
Abstracts that you have prepared							
Editorials that you have written							
Submitted case reports/continuous medical education material							
Number of articles that you have been asked to review by editorial boards during COVID-19							
Compared to before the pandemic	More			Less		Same	
Are the above activities more or less?							
Is the number of webinars that you have attended/provided more or less?							
Is the number of online courses/e-learning that you have completed more or less?							

The questionnaire was distributed via WhatsApp at the national level. Responses were collected over a one-month period in June 2020 (three months after the announcement of the COVID-19 pandemic). The reliability of the questionnaire was estimated using a test-retest approach with 10 respondents. Cohen’s κ was calculated, indicating moderate agreement with 87.5% agreement and Cohen’s κ 0.6, for which the questionnaire was considered reliable.

The academic productivity of each HCP was assessed individually. The number of publications, such as original articles, abstracts, editorials, and case reports, was calculated. Attendance at webinars and online courses was also recorded.

Statistical analysis

Data were statistically analyzed using SPSS Statistics version 24 (IBM Corp. Released 2016. IBM SPSS Statistics for Windows, Version 24.0. Armonk, NY: IBM Corp.). Frequency and percentages were calculated for categorical variables. Responses related to scientific productivity were compared between doctors and nurses using Fisher’s exact test. Responses related to academic and educational activities before and after the COVID-19 pandemic were compared between doctors and nurses using Pearson’s chi-square test. P-value <0.05 was considered statistically significant.

Ethical approval

This study was ethically approved by the Secondary Health Care Research Committee (SHCRC), Salmaniya Medical Complex, Ministry of Health, Kingdom of Bahrain (IRB number: 69060521). It was also performed in accordance with the Declaration of Helsinki. All the participants gave consent upon filling out the questionnaire.

## Results

**Figure 1:**
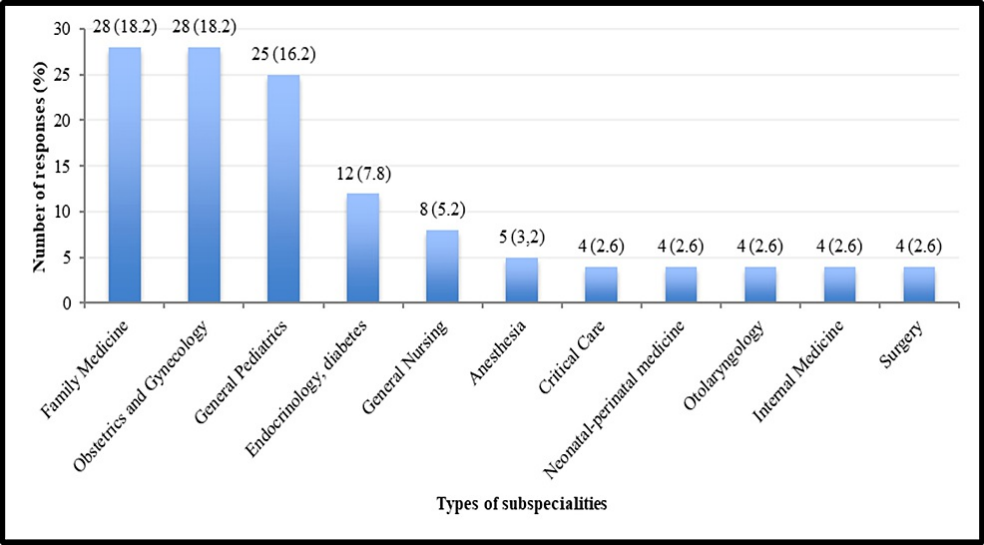
Number and percentage of responses among different subspecialties (n=154). The remaining 29 responses came from other subspecialties (≤3 responses).

Responses were received (100 (64.9%) doctors and 54 (35.1%) nurses) with an overall response rate of 75.5%. All 154 participants were included in the study. Out of the 100 doctors, 58 (58%) were consultants, 21 (21%) were specialists, and 21 (21%) were residents. All the HCPs included in the study belonged to the government health sector. The participants were working in different medical specialties (Figure 1).

Most responses came from obstetricians and gynecologists (28 (18.2%)) and family physicians (28 (18.2%)), followed by pediatricians (25 (16.2%)). Three responses came from gastroenterologists, infectious disease specialists, diagnostic radiologists, hematologists, and oncologists; two were from allergists, cardiologists, dermatologists, emergency medicine specialists, geriatric medicine specialists, ophthalmologists, orthopedic surgeons, and psychiatrists.

Academic activities

**Figure 2:**
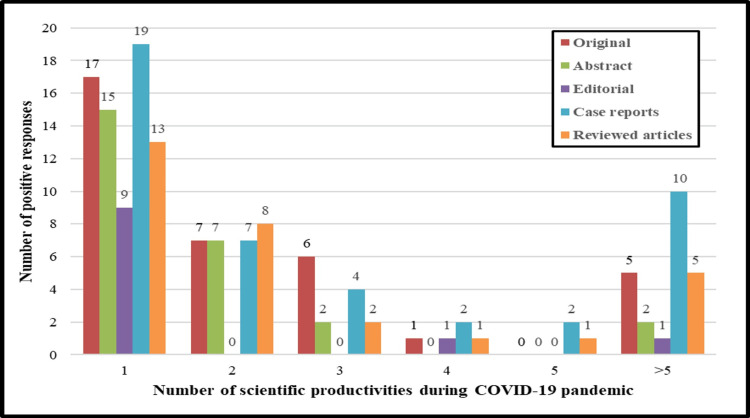
Number of scientific productivities during the COVID-19 pandemic among healthcare professionals.

Most of the Bahraini doctors and nurses could not prepare any editorials (94 (94%) doctors versus 49 (90.7%) nurses) or abstracts (83 (83%) doctors versus 45 (83.3%) nurses) during the studied period of the pandemic (Figure 2).

However, 24 (24%) of doctors and 12 (22.2%) of nurses managed to submit at least one original article for publication (Table 2).

**Table 2:** Comparison between healthcare professionals on the impact of COVID-19 on educational and academic activities.

Scientific productivity	Profession		Total, n (%)	P-value
	Doctors, n (%) n = 100 (64.9)	Nurses, n (%) n = 54 (35.1)	n = 154 (100)	
Submitted at least one original article	24 (24)	12.0 (22.2)	36 (23.4)	0.845
Submitted at least one abstract	17 (17)	7.0 (13)	24 (15.6)	1.000
Wrote at least one editorial	6.0 (6.0)	5.0 (9.3)	11 (7.1)	0.518
Submitted at least one case report and continuous medical education material	25 (25)	19 (35.2)	44 (28.6)	0.195
Reviewed at least one article by editorial boards	16 (16)	14 (26)	30 (19.5)	0.143

The majority of submissions came from consultants (17%), compared to specialists (5%) and residents (2%). Moreover, 19 (35.2%) of nurses were able to submit at least one case report or continuous education material versus 25 (25%) of participating doctors (18% of the consultants, 4% of the specialists, and 3% of the residents). Furthermore, 14 (26%) of nurses were asked by the editorial boards of journals to review articles in comparison to 16 (16%) of doctors (Table 2). Nevertheless, in all five questions, there was no statistically significant difference between doctors and nurses.

Educational activities

Both doctors and nurses stated that their overall academic activities were less compared to the time before the pandemic, yet this was not statistically significant (P=0.059), as shown in Table 3.

**Table 3:** Comparison of the impact of the COVID-19 pandemic on the educational and academic activities of healthcare professionals prior to the pandemic.

Academic/educational activities	Profession		P-value
	Doctors, (n = 100)	Nurses, (n = 54)	
Overall academic activities status compared to prior to pandemic			0.059
Less	74 (74)	32 (59.2)	
More	26 (26)	22 (40.8)	
Webinars attended compared to prior to pandemic			0.022
Less	33 (33)	29 (54)	
More	54 (54)	17 (31.5)	
Same	13 (13)	8.0 (14.5)	
Online courses attended compared to prior to the pandemic			0.014
Less	30 (30)	28 (52)	
More	47 (47)	21 (39)	
Same	23 (23)	5.0 (9.0)	
Values are presented as numbers (%). P-values <0.05 were statistically significant			

Webinars and online courses were attended more by doctors and less by nurses compared to the time before the pandemic (P=0.022, P=0.014, respectively).

## Discussion

As the outbreak of COVID-19 has evolved into a worldwide pandemic, the services provided by healthcare systems across the globe have been widely affected. The health crisis caused by COVID-19 has resulted in a shift in the priorities of healthcare systems toward containing the virus's spread and reducing its’ related mortality and morbidity [8]. For instance, elective surgical procedures were canceled, and telehealth was used instead of clinic visits in order to reduce exposure, prevent the spread of the infection, and reallocate resources towards more critical areas [9,10]. Similarly, academic activities were affected remarkably during the pandemic. A study conducted in Europe in October 2020 showed that the average time spent on educational activities such as journal clubs and grand rounds was around 9.9 hours per week before the pandemic. This time was reduced to 4.7 hours per week after the pandemic started [9].

In this study, both doctors and nurses in Bahrain felt that their overall engagement in academic activities during the COVID-19 pandemic was less compared to the time before the pandemic (74 (74%) and 32 (59.2%), respectively). This is comparable to international figures, where 69% of doctors and 62% of nurses had a similar impression [7]. This finding can be attributed to a number of factors, the most apparent being the increasing physical workload on HCPs during the pandemic, resulting in less time to spare on educational activities [11,12]. Furthermore, the variable psychological implications and mental challenges HCPs were obliged to face during the pandemic hindered their ability to shift focus toward academic and educational events [12-14]. Moreover, other challenges affect HCPs' participation, such as a lack of computer proficiency, isolation from colleagues, and limited opportunities for free discussion.

In the current study, doctors, contrary to nurses, reported a lesser number of case report submissions, article reviews, and editorial preparations during the pandemic. For instance, 94% of doctors, compared to 90.7% of nurses, did not prepare any editorials during this period. On the other hand, nurses reported a lesser number of original article submissions when compared to doctors (22.2% of nurses submitted at least one original article during the pandemic versus 24% of doctors). This reduction in educational activities has been correspondingly noted in several studies. For example, Amparore et al. found that the proportion of residents who reported a reduction or complete suppression of educational and training activities reached up to 81.2% of the 351 residents involved in their study [15].

Remarkably, this study showed that residents participated less in educational activities in comparison to consultants during the crisis. Furthermore, only two residents submitted at least one original article, compared to 17 consultants and five specialists. Isa et al. reported the same in their study, which was conducted prior to the pandemic. He stated that 76.6% of pediatricians who published scientific papers were consultants, and only 23.4% were residents [16]. This might be explained by the fact that the residents are busier preparing for their board exams and doing in-house overnight calls on a regular basis compared to consultants who are doing on-call duties from home. Moreover, residents might lack the ability to conduct research and the awareness of the importance of research compared to consultants who need the research for their academic development, as many of them are part-time lecturers or professors in medical colleges. Therefore, we recommend giving the residents spare time or protected time for research and involving them in research-conducting workshops.

On the other hand, as a result of the limitations imposed by the COVID-19 pandemic, especially with regard to the restrictions on the congregation, there is a rapid move globally toward the use of digital technology in order to ensure the continuity of medical and academic activities [11]. Methods being used include, but are not limited to, online courses, webinars, and the utilization of social media applications [11]. Due to the feasibility, time-saving, and cost-effectiveness of these methods, there is a promising likelihood of their continued use in the post-COVID era [17].

In our study, despite the fact that both nurses and doctors reported overall less involvement in academic activities during the pandemic, more than half of the doctors reported increased attendance at webinars and online courses. Likewise, an interventional endoscopy center based in Orlando, United States of America, reported that although they had fewer patients for clinical trials during the COVID-19 pandemic, there was a surge in academic productivity in 2020 when compared to the previous year. This increase was attributed to the transformation from life to online courses, resulting in more attendees with less cost [18].

However, many challenges to online medical education during the COVID-19 pandemic have been reported. For instance, Rajab et al. found in their study that issues related to communication, use of technology tools, online experience, pandemic-related anxiety or stress, and technophobia have an effect on online education [19]. Furthermore, Shetty et al. reported that a sedentary life with decreased outdoor activity and eye-related problems like eye strain, epiphora, and headache were major disadvantages of online education [20]. There are some limitations to this study. For instance, it has been conducted in a relatively short period after the start of the pandemic; it documents a short-term impact; and a long-term study will be of definite value. As the survey was conducted online, its’ accessibility was limited to those who are active online. Another limitation is that the survey did not include details of the scientific productivity of the HCPs prior to the pandemic. It is important to have objective evidence about the impact of the pandemic on HCPs' scientific performance. In this survey, we relied on HCPs' assessments of their own performance in comparison to the period prior to the pandemic. This way of collecting data might be subjected to a recall bias, but that could not be avoided. Furthermore, we should have asked the respondents about the possible solutions to overcome the challenges they faced during the pandemic and improve their performance. Despite these limitations, the results of this study are important to understand the impact of the COVID-19 pandemic on the academic development of HCPs, which can subsequently affect the medical care of patients.

## Conclusions

In our study, overall academic and educational activities were reduced during the COVID-19 pandemic compared to before the pandemic. However, online education is a growing field that has expanded during the pandemic. There is a rapid move in the direction of the use of digital technology globally, as it is more practical, cost-effective, efficient, and time-saving. There is a promising likelihood of their continuity in the post-COVID era.

Running another study to understand the reasons for this reduction is recommended, and studying the factors that could sustain and promote educational activities during pandemics is desirable.
